# Toxicological Risk Assessment of Coffee Oil (Coffee Seed Oil and Spent Coffee Grounds Oil) as a Novel Food with Focus on Cafestol

**DOI:** 10.3390/molecules30142951

**Published:** 2025-07-12

**Authors:** Bernadette Maier, Heike Franke, Steffen Schwarz, Dirk W. Lachenmeier

**Affiliations:** 1 Postgraduate Study of Toxicology and Environmental Protection, Rudolf-Boehm-Institut für Pharmakologie und Toxikologie, Universität Leipzig, Härtelstrasse 16-18, 04107 Leipzig, Germany; cr16ixig@studserv.uni-leipzig.de (B.M.); heike.franke@medizin.uni-leipzig.de (H.F.); 2 Chemisches und Veterinäruntersuchungsamt (CVUA) Karlsruhe, Weissenburger Strasse 3, 76187 Karlsruhe, Germany; 3 Coffee Consulate, Hans-Thoma-Strasse 20, 68163 Mannheim, Germany; schwarz@coffee-consulate.com

**Keywords:** coffee oil, novel food, cafestol, kahweol, *Coffea arabica*, *Coffea canephora*, risk assessment, toxicological review

## Abstract

Coffee oil derived from spent coffee grounds of *Coffea arabica* is considered a novel food in the European Union (EU), requiring pre-market approval supported by comprehensive toxicological data. The effects of coffee oil on human health, particularly on blood parameters and liver enzymes, have been investigated in several studies. This review article summarizes the available toxicological literature on coffee oil, including its bioactive diterpenes cafestol and kahweol, which are known for their potential health effects. Considering the different modes of action of these two diterpenes, moderate consumption of coffee oil may be considered safe for healthy adults. Based on the changes in serum values in humans, this review provides initial estimations of LOAEL, NOAEL, and ADI for these diterpenes. The findings suggest that an intake of 225 mg of coffee oil per day might be considered safe assuming that coffee oil contains about 0.4% diterpenes. In summary, the assessment based on the published data indicates that (i) the consumption of coffee oil contained in any type of prepared coffee appears to be safe because the homeostasis of lipid levels in the blood is not significantly affected, and (ii) a low consumption of coffee oil as such might be acceptable but would require a refined risk assessment considering the exposure levels of the intended food product, which must be provided for novel food approval procedures.

## 1. Introduction

**Figure 1:**
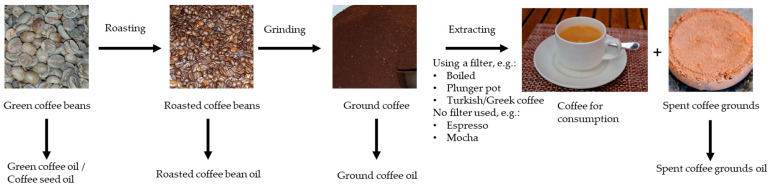
Sources of coffee oil (sources: own photographs).

Botanically, coffee belongs to the Rubiaceae family and is classified under the genus *Coffea* [1,2,3]. *Coffea arabica* (Arabica) and *Coffea canephora* (Robusta) are the most commonly consumed coffee species, with *Coffea arabica* accounting for around 63% of the total coffee production for green beans for the first six months of coffee year 2024/25 to March 2025 [3,4,5]. In March 2025, according to the International Coffee Organization, 0.78 Mio tons of green coffee beans were produced in total. The top producers of all forms of coffee are from South America followed by Asia and Oceania [4]. The USDA has made the forecast that in 2024/25, coffee production will be about 10.6 Mio tons [6]. According to Eurostats, 2.7 Mio tons of coffee was imported into the European Union (EU) in 2023. Of these, Germany imported around 33% (911,300 tons), followed by Italy with 23% (624,600 tons) and Belgium with 10% (278,200 tons) [7]. Coffee beans contain lipids, known as coffee oil, which can be extracted either directly from the unroasted green bean (coffee seed oil), the roasted bean, either directly or after grinding (roasted (ground) coffee seed oil), or from the spent roasted coffee grounds following coffee extraction (spent coffee grounds oil) (Figure 1).

In green coffee beans, lipids are embedded in the cytoplasm of the native plant cell within separate membrane-protected oil bodies located along the cell walls. The lipids are mainly found in the endosperm of the green coffee, and a small amount, the coffee wax, is located in the outer layer of the bean. During roasting, the cells are destroyed, which mobilizes the coffee oil. While roasting, so-called “oil sweating” is sometimes observed, caused by the gas pressure inside the bean, which pushes the coffee oil to the bean surface [8,9,10].

Green coffee oil, the coffee oil extracted from raw coffee beans, is usually greenish yellow with a slight odor. Coffee oil, derived from roasted coffee samples (roasted coffee oil, grounds coffee oil, and spent coffee grounds oil), is a brown viscous liquid and has an aroma that is mainly derived from products of the Maillard and Strecker reactions that occur during the roasting process [8,10,11,12,13].

Depending on the analytical methods used, the lipid content of coffee beans ranges from 8 to 17% [9,13,14]. *Coffea canephora* generally contains less lipids (around 10%) than *Coffea arabica* (around 15%) [9]. Another equally significant factor regarding the lipid content is whether raw coffee beans, roasted coffee beans, or spent coffee grounds are analyzed [13]. In general, it is observed that green coffee beans contain about 0.4–4% less lipids than the corresponding roasted beans (to be explained by the mass loss of water of about 12–20% during roasting), but, e.g., the composition of fatty acids only changes slightly [8,10,14].

Unlike spent coffee grounds, defatted spent coffee grounds, and defatted unused coffee grounds themselves [15], the isolated coffee grounds oil is classified as a novel food in the European Union (EU) because it has not been consumed to a significant degree as such in the EU before 15 May 1997 [16] (similarly, the pure coffee silver skin is classified as a novel food [17,18]). While not specifically mentioned in the novel food consultation response [16], it can be assumed that all forms of coffee oil must be treated as novel food for the same reason.

As a novel food, coffee oil therefore needs a pre-market authorization before being placed on the market within the EU. The authorization procedure also includes a risk assessment by the European Food Safety Authority (EFSA). Considering the necessary data for the authorization procedure, this review article summarizes the available toxicological literature on coffee oil, including cafestol and kahweol [19,20], and concludes with a judgement about possibly missing data according to the EFSA guidelines.

## 2. Literature Research

For this article, electronic literature searches were performed using various databases, including SciFinder^n^ (American Chemical Society, Columbus, OH, USA), PubMed (National Library of Medicine, Bethesda, MD, USA), and Google Scholar (Google LLC, Mountain View, CA, USA). The search results were used to trace the publication history on the topic, and authors with relevant works were further investigated through Google (Google LLC, Mountain View, CA, USA) and ResearchGate (Research-Gate GmbH, Berlin, Germany) to review their publication lists for additional and more recent articles. The outcome was a narrative review that aimed to comprehensively cover the available evidence.

A broad array of search terms was employed for a systematic search of toxicological literature, including terms such as coffee oil, cafestol, kahweol, coffee oil diterpenes, and coffee (limited to toxicological findings). Additionally, databases such as the European Food Safety Authority (EFSA) database on food intake were explored for data on the respective topics.

## 3. Compositional and Toxicological Data on Coffee Oil

### 3.1. General Composition of Coffee Oil

The main components of coffee oil from the green beans are triacylglycerols and unsaponifiable matter, such as cafestol, kahweol, and their esters, but also sterols, tocopherols, phosphatides, tryptamine derivates, and other minor components. The insights regarding the lipid fraction of the coffee bean were investigated in detail by Speer and Kölling-Speer [9]. The composition of lipids in green coffee is summarized in Table 1.

#### 3.1.1. Triacylglycerols

The triacylglycerols with around 75% vary from C12 to C24 saturated and unsaturated carbon chains and their respective derivatives, such as esters or hydrolyzed products. The main fatty acids are palmitic acid (C16:0, 14.1–42%) and linoleic acid (C18:2, 25.5–54.3%) [9,14,21,22,23,24]. In summary, the profile of fatty acids is similar to common edible vegetable oil [9,25] and does not appear to pose a toxicological risk. Oliveira et al. suggested the use of crude coffee oil in the food and pharmaceutical industries, but the content of unsaponifiable matter and free acidity should be lowered with refining of the oil [25].

#### 3.1.2. Diterpenes and Their Derivatives

**Figure 2:**
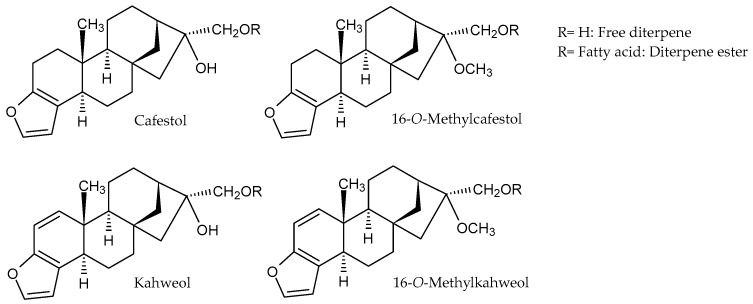
The chemical structure of diterpenes from coffee oil.

A notable difference in coffee oil compared to other vegetable oils is the presence of the diterpene alcohols cafestol and kahweol, as well as 16-*O*-methylcafestol and 16-*O*-methylkahweol and their respective ester derivatives, which are together part of the unsaponifiable fraction of coffee oil (Figure 2) [9,25]. Within *Coffea* plants, cafestol and kahweol compounds show anti-fungal and anti-insect activities [26]. The structure of these diterpenes consists of pentacyclic diterpene alcohols based on the kauran structure. Cafestol and kahweol are both sensitive to acids, heat, and light, whereas kahweol is especially unstable in its purified form [9,27]. Because of these characteristics, diterpenes are more often present in the form of esters than as free diterpene alcohols (Table 1).

The first research and subsequent structural clarification of cafestol and kahweol was performed by several workgroups between 1932 and 1960, e.g., Bengis et al., Chakravorty et al., Wettstein et al., and Haworth and Finnegan et al. [10,11,12,13,14,15]. The isolation of 16-*O*-methylcafestol was initially performed in 1989 by Speer et al. [28]. Later, in 2001, 16-*O*-methylkahweol was isolated by Kölling-Speer and Speer from beans of *Coffea canephora* [9].

#### 3.1.3. Distribution of Diterpenes in *Coffea arabica* and *Coffea canephora*

Green coffee beans typically have a higher content of diterpenes than their roasted counterpart [29]. It is notable that *Coffea arabica* contains free cafestol as well as free kahweol and corresponding fatty esters [9,30]. For a long time, it was believed that the beans of *Coffea arabica* do not contain 16-*O*-methylcafestol. 16-*O*-Methylcafestol was only found in other parts of the coffee plant, e.g., in the leaves [9,31]. In 2018, using more sensitive methods, Gunning et al. showed that 16-*O*-methylcafestol had not been found in *Coffea arabica* because in the past, it had been below the analytical threshold. In fact, *Coffea arabica* contains trace amounts of 16-*O*-methylcafestol, as detected via NMR [24,32,33] and UPLC-MS/MS experiments [34]. However, this does not hinder the use of this compound as a marker for coffee authentication purposes in commercial products [35]. *Coffea canephora* contains free cafestol as well as fatty acid esters thereof. Additionally, it entails small amounts of 16-*O*-methylcafestol and only trace amounts of kahweol and 16-*O*-methylkahweol [9,30,31,36,37]. To the best of the authors’ knowledge the presence of cafestol and kahweol in other plant species besides *Coffea* is not reported [38]. In the following review, esterified and free cafestol/kahweol compounds will not be distinguished as ester cleavage usually takes place during digestion. For quantitative reasons, the authors presume in the following that coffee oil from *Coffea arabica* contains cafestol as well as kahweol, and *Coffea canephora* contains only cafestol.

### 3.2. Coffee Oil Extraction

Generally, the coffee oil yield depends on the botanical coffee species and also on the quality of the bean, the particle size, extraction method, extraction time, and solvent [12]. The different extraction methods and analytical systems, such as gas chromatography or liquid chromatography with their different detectors, show slightly different results for the composition of coffee oil, which also differs with different *Coffea* species [14]. There are various ways to extract coffee oil from the beans, with the most commonly used methods being pressing, Soxhlet extraction, microwave-assisted extraction (MAE), and supercritical fluid extraction (SFE) [12,29].

In the industry, pressing is traditionally the mostly used mechanical extraction method, which is solvent-free and therefore a green and sustainable approach, but it also results in lower yields and purity than other methods [12,39]. The Soxhlet extraction method is especially used in the laboratory when detailed and accurate analyses are needed. Although Soxhlet extraction is time-consuming and environmentally problematic due to the high usage of organic solvents, it provides a good purity of the resulting coffee oil [12]. MAE is a fast method that has a less negative impact on the environment, as the method reduces the use of organic solvents. Yet, challenges arise due to extraction uniformity and equipment cost [12]. When using CO_2_ as an extraction medium, SFE is an efficient and environmentally friendly method, as CO_2_ derives directly from the gas discharge from the industry or even from the air (following concepts such as Industry 4.0 or Circular Economy). With SFE, high-purity coffee oil can be gained, although the initial cost of the equipment is high [12,40]. There are hundreds of patents dealing with SFE as an extraction method in food processing, and it is industrially used, e.g., for coffee decaffeination [41].

### 3.3. Potential Use of Coffee Oil in Food Products

At the moment, coffee grounds are a waste product of the soluble coffee industry and are generated in a wide variety of sectors, e.g., the domestic, commercial, or industrial sector, specifically the manufacture of coffee extracts. Following beverage preparation, spent coffee grounds are typically discarded directly into waste receptacles, subsequently transported to landfills where they generate greenhouse gases such as methane and CO_2_, or alternatively, directed toward energy recovery applications. Alternative uses can be found in the areas of solid fuels, fertilizers, or as additives for animal feed. Coffee grounds can also be used for composting and for soil improvement [42,43]. Franca et al. summarized different potential application areas of spent coffee grounds, such as energy (e.g., solid fuels, biofuels), chemicals (e.g., cosmetics, surfactants), materials (e.g., flame retardants, thermal insulation), food-related applications such as food ingredients (e.g., peptides, antioxidants), as food packaging (e.g., biopolymers), or for food products [42]. All these applications could be a practical and innovative idea in order to increase the overall sustainability of the coffee agro-industry [42,44,45,46,47,48].

Ribeiro et al. suggested the use of coffee oil gained from roasted beans in candies, chocolates, ready-to-eat drinks, for gourmet applications, and for instant coffee aromatization due to the pleasant roasted coffee aroma [12]. To the best of the authors’ knowledge, so far, five authors have addressed the application of coffee oil itself in the food sector [49,50,51,52,53].

Deotale et al. suggested the use of coffee oil as a natural surfactant (oil-in-water emulsifying agent) in foods, cosmetics, and in pharmaceutical applications [49]. Meerasri et al. partially substituted up to 30% of butter with coffee oil and observed an increase in the total phenolic content and antioxidant properties. Nevertheless, with higher amounts of coffee oil (>20%), the flavor rating decreased due to the smell of coffee oil from spent coffee grounds, which led to an undesirable mouthfeel [50]. Coffee oil might also be suitable to replace synthetic antioxidants in order to extend the shelf life of refined sunflower oil. Based on different stability indices, Bijla et al. suggested that the use of 0.03% of an ethanolic spent coffee grounds extract did not have any negative effect on the flavor of sunflower oil [51]. De Oliveira et al. reported the use of a microcapsule application of up to 0.69% (*w*/*v*) green coffee oil in tamarind juice aiming to avoid oxidation processes [52]. Frascareli et al. reported that coffee oil is used to minimize the amount of powder in soluble coffee as the oil forms a superficial layer and prevents the fragmentation of the soluble coffee grains. Moreover, the authors reported on the microencapsulation of coffee oil in order to avoid degradation processes if used in foods, e.g., for aromatizing soluble coffee and coffee drinks as well as for flavoring sweets, cakes, and puddings [53].

Based on the possibility of increasing the stability of foaming [49], further possible applications in the food sector are conceivable, where soft and liquid foams are desired, e.g., on top of beverages or for desserts such as whipped cream, mousse, ice cream, or others [54]. As shown before, coffee oil has the potential to be used as a food additive [49,50,51]; therefore, other applications might be imaginable, e.g., in salad oil, cakes, or tiramisu.

### 3.4. Influence of the Brewing Technique on Coffee Oil in Coffee Beverages

Urgert et al. summarized several brewing techniques for coffee, such as filtered, percolated, and instant coffee, as well as espresso, mocha, boiled, blunger pot, and Turkish/Greek coffee. According to this study, techniques that do not require filtering, such as boiled, blunger pot, and Turkish/Greek coffee, lead to noticeably higher concentrations of cafestol and kahweol in the extracted beverage than the other techniques [55]. Ahola et al. described that more than 80% of the fatty compounds were retained by a paper filter [56]. Furthermore, Buchmann et al. showed that the preparation parameters of coffee, such as the coffee/water ratio, water temperature, or particle size, also have an influence on the cafestol content in coffee [57]. Which form of coffee brewing technique is preferred relates to the country of origin but also varies between individual preferences, which can change over time [55]. Although coffee oil is poorly soluble in water, the lipids could be incorporated in the brew depending on the method of infusion [9]. In a detailed literature review, Moeenfard et al. summarized that, with regard to the brewing process, the highest and lowest cafestol and kahweol contents were found in boiled/Turkish coffee (22–138 mg/mL) and instant/filtered coffee (0.5–2.3 mg/L), respectively [58].

Spent coffee grounds also contain coffee oil, as not all of the coffee oil gets into the beverage or remains on the filter. An important aspect is that the chemical profile of the different sources of coffee oil is always about the same. Therefore, the coffee oil composition directly derived from green or roasted beans is similar to coffee oil originating from coffee grounds. In this context, it should also be noted that this is also the case for coffee beans obtained from different countries around the world, possibly related to the rather restricted genomic variability in commercial coffee varieties [44,45].

### 3.5. Estimated Intake of Cafestol and Kahweol

Coffee oil containing cafestol and kahweol is predominantly ingested with unfiltered beverages, e.g., boiled coffee, fully automated piston-type coffee, or espresso. By drinking unfiltered coffee beverages such as Turkish coffee, coffee grounds are also partially consumed as part of the sediment [59,60]. However, filtered coffee also contains a certain amount of coffee oil, but much less than unfiltered coffee [21,61].

While Urgert et al. found up to 3.9 mg cafestol/150 mL cup and 4.4 mg kahweol/150 mL cup [55] in boiled, blunger pot, and Turkish/Greek coffee as the preparation method, Buchmann et al. detected up to 2.7 mg cafestol/90 mL cup espresso by varying the preparation parameters, e.g., the coffee/water ratio, water temperature, and particle size [57].

Ranheim et al. summarized that filtered coffee brews generally contain less diterpenes than unfiltered beverages, e.g., Scandinavian boiled, Turkish/Greek, French press, or espresso (Table 2) [62]. As there is no standardization in brew preparation, the cafestol and kahweol contents show wide variability [63].

In the most extreme case, 100 mL of Scandinavian brewed coffee could contain up to 18 mg of diterpenes (8 mg/100 mL cafestol and 10 mg/100 mL kahweol) (see Table 2). Estimating daily coffee consumption is challenging due to numerous influencing factors. Due to the lack of reliable data for the EU, this study adopts a daily maximum of 1914 mL of coffee per capita, based on the 2023 findings of Konstantinidis et al. using data from Denmark [64]. In accordance with these assumptions, it could be assumed that the worst-case maximum daily intake of the diterpenes cafestol and kahweol might be about 6 mg diterpenes/kg bw/day (Table 3).

### 3.6. Bioavailability of Cafestol

Earlier studies reported that the absorption of cafestol, administered as its palmitate, is about 70% at the intestinal level, while 20% is degraded in the stomach environment [65,66,67], but a new study by Brand et al. shows that the bioaccessibility of cafestol from boiled coffee brew is about 94%, which was shown using an in vitro digestion model. Around 67% of cafestol was absorbed into the duodenum, 24% was lost in the stomach, and eight hours after ingestion, 1% was excreted in the urine as sulfate or glucuronide conjugates [63]. Additionally, Urgert et al. described that about 5% of diterpenes are recovered in the feces [31]. These findings should be observed in contrast to the low bioavailability (about 13%) of cafestol and kahweol when spent coffee grounds are consumed directly. Herein, most of the diterpenes are excreted in feces [29,68].

In addition to human studies, it is recommended to use special animal models, e.g., the ApoE*3-Leiden transgenic mouse that responds to cafestol as humans do, especially when studying the body distribution, portal bioavailability, and biliary excretion of cafestol. ApoE*3-Leiden transgenic mice are therefore a preferrable model to investigate the pharmacokinetic and pharmacodynamic effects of cafestol and to draw conclusions about the mechanistic processes in the human body [69,70]. A study from Van Cruchten et al., using ApoE*3-Leiden transgenic mice, examined in more detail the whole-body distribution, biliary excretion, and portal bioavailability of cafestol in mice [69]. Using ^3^H-labeled cafestol, it was shown that five hours after oral administration, most activity was found in the small intestine, liver, and bile. This indicates that in these organs, almost all of the cafestol metabolites accumulate, whereas hardly any distribution to other parts of the body was found. Further experiments, e.g., with radiolabeled cafestol, revealed that the compound was fully eliminated 48 h after the oral dose [69]. The study confirmed that cafestol mostly undergoes enterohepatic cycling: cafestol is extensively metabolized in a phase II biotransformation process by the liver to epoxyglutathione conjugates, glutathione conjugates, and glucuronide conjugates, which are subsequently excreted into the bile [29,69,71].

### 3.7. Human Studies

#### 3.7.1. Epidemiology

As early as 1985, a study by Førde et al. revealed that humans who drank boiled coffee instead of filtered coffee showed an increase in serum cholesterol concentrations [72]. With this publication, an explanation was given for the partly contradictory findings that had preceded it. Herein, some authors found the link between coffee consumption and higher serum cholesterol concentrations, whereas others did not or could not exclude other possible confounding factors including smoking, age, sex, or locality [73]. Subsequent studies confirmed the findings of Førde et al. [72], which proved that the consumption of boiled, unfiltered coffee and the rise in serum cholesterol, especially the rise in cholesterol, may be causally related [56,73,74,75,76,77,78,79]. The mechanism behind these effects involves alterations in lipid metabolism. Research indicates that cafestol raises the activity of cholesterylester transfer protein and phospholipid transfer protein by approximately 18% and phospholipid transfer protein by about 21% while reducing lecithin:cholesterol acyltransferase activity by 11%. These changes help to explain how coffee diterpenes influence serum lipid levels [80].

Tverdal et al. [81] combined different cohort studies from Norway using data from 1985 to 2003 with a total of 635,718 men and women aged 20 to 79 years. The link between the brewing method of the coffee (filtered vs. unfiltered) could be explained by the lipid-raising effect of the diterpenes in unfiltered coffee. It was concluded that there is a higher total and cardiovascular disease (CVD) mortality risk for people who consumed unfiltered coffee than filtered coffee. Consumers of one to four cups of filtered coffee per day showed the lowest mortality [81]. It should be added that there are also contrary findings concerning the rise in serum cholesterol even if paper filters are used. This could be explained by reports showing that diterpenes are only partly removed by paper filters [82].

There are multiple positive as well as negative effects on human health associated with the consumption of coffee containing cafestol and kahweol [58]. The increase in serum cholesterol, triacylglycerides, and LDL (low-density lipoprotein) may contribute to coronary health risks, for example, myocardial and cerebral infarction, insomnia, cardiovascular complications, aortic valve stenosis, and thrombosis [58,83,84]. Urgert et al. assumed that an increase in cholesterol concentration of 6–10% may increase coronary risk by 12–20%, especially for younger people [77]. However, it is important to consider that confounding variables might affect this relationship.

#### 3.7.2. Effect on Serum Lipids

**Figure 3:**
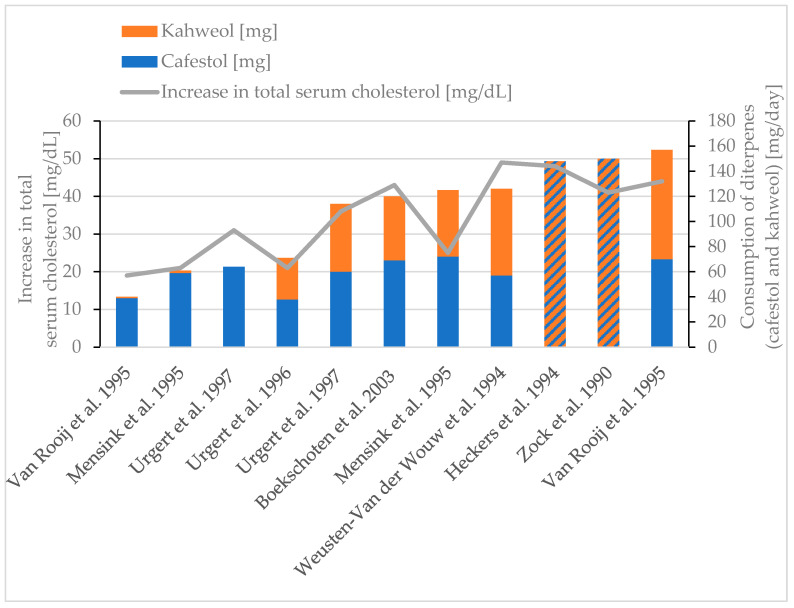
Increase in total serum cholesterol. Review of eight studies [30,38,77,78,85,86,87,88]. Striped bars (diagonal hatching): only the diterpene content is given in the respective study [78,88].

Studies were reviewed regarding the effects on blood serum lipids if diterpenes or coffee oil is consumed (Table 4). Cholesterol, produced by liver cells, is released into the bloodstream, known as serum cholesterol. A higher intake of diterpenes results in an increase in total serum cholesterol [30,38,77,78,85,86,87,88] (Figure 3). Although the intakes during the studies are way below the estimated maximum daily intake of 345 mg of diterpenes (153 mg of cafestol and 191 mg of kahweol, Table 3) if high amounts of unfiltered coffee are consumed, Urgert et al. estimated that each 10 mg of cafestol ingested per day raises the total serum cholesterol by 0.15 mmol/L (equals 5.8 mg/dL) [31].

Generally, the serum cholesterol of a healthy human is around 220–280 mg/dL, while higher values are common at an advanced age, especially for women [89]. The studies considered show an increase in total serum cholesterol depending on the diterpene content of the beverage, which could lead to undesirably high total serum cholesterol levels possibly outside the homeostatic range.

**Table 4:** Serum lipids: examples of effects in humans due to diterpene (cafestol/kahweol) consumption.

Effect	Studies with Humans	Cafestol/Kahweol Content (per Day)	∆
Rise in total serum cholesterol	Boekschoten et al., 2003 [86] 2 mL coffee oil/day, 5 weeks	69 mg cafestol and 51 mg kahweol	+43 mg/dL
	Weusten-Van der Wouw et al., 1994 [38] 2 g coffee oil/day, 4 weeks	57 mg cafestol and 69 mg kahweol	+49 mg/dL
	Heckers et al., 1994 [88] 148 mg diterpenes/day	N/A	+48 mg/dL
	Van Rooij et al., 1995 [30] 1 g coffee oil (Arabica/day), 6 weeks	70 mg cafestol and 87 mg kahweol	+44 mg/dL
	Van Rooij et al., 1995 [30] 1 g coffee oil (Canephora/day), 6 weeks	39 mg cafestol and 1 mg kahweol	+19 mg/dL
	Urgert et al., 1997 [85] 64 mg cafestol/day, 28 days	64 mg cafestol	+31 mg/dL
	Urgert et al., 1997 [85] 114 mg diterpenes/day, 28 days	60 mg cafestol and 54 mg kahweol	+36 mg/dL
	Urgert et al., 1996 [77] 71 mg diterpenes/day, 24 weeks	38 mg cafestol and 33 mg kahweol	+21 mg/dL
	Mensink et al., 1995 [87] 2 g coffee oil/day, 3 weeks	72 mg cafestol and 53 mg kahweol	+25 mg/dL
	Mensink et al., 1995 [87] 2 g coffee oil/day, 3 weeks	59 mg cafestol (cafestol and *O*-methyl-cafestol) and 2 mg kahweol	+21 mg/dL
	Zock et al., 1990 [78] 1.3 g coffee lipids/day, 6 weeks	150 mg unsaponifiable lipids	+41 mg/dL
Rise in triacylglycerol	Boekschoten et al., 2003 [86] 2 mL coffee oil/day, 5 weeks	69 mg cafestol and 51 mg kahweol	+64 mg/dL
	Weusten-Van der Wouw et al., 1994 [38] 2 g coffee oil/day, 4 weeks	57 mg cafestol and 69 mg kahweol	+73 mg/dL
	Van Rooij et al., 1995 [30] 1 g coffee oil (Arabica/day), 6 weeks	70 mg cafestol and 87 mg kahweol	+72 mg/dL
	Urgert et al., 1997 [85] 64 mg cafestol/day, 28 days	64 mg cafestol	+58 mg/dL
	Urgert et al., 1997 [85] 114 mg diterpenes/day, 28 days	60 mg cafestol and 54 mg kahweol	+63 mg/dL
	Heckers et al., 1994 [88] 148 mg diterpenes/day	N/A	+65 mg/dL
	Urgert et al., 1996 [77] 71 mg diterpenes/day, 24 weeks	38 mg cafestol and 33 mg kahweol	+25 mg/dL
	Mensink et al., 1995 [87] 2 g coffee oil/day, 3 weeks	72 mg cafestol and 53 mg kahweol	+48 mg/dL
	Mensink et al., 1995 [87] 2 g coffee oil/day, 3 weeks	59 mg cafestol (cafestol and *O*-methyl-cafestol) and 2 mg kahweol	+43 mg/dL
	Zock et al., 1990 [78] 1.3 g coffee lipids/day, 6 weeks	150 mg unsaponifiable lipids	+45 mg/dL
No significant effect on triacylglycerol	Van Rooij et al., 1995 [30] 1 g coffee oil (Canephora/day), 6 weeks	39 mg cafestol and 1 mg kahweol	No significant change
Decrease in HDL cholesterol	Urgert et al., 1997 [85] 64 mg cafestol/day, 28 days	64 mg cafestol	−2 mg/dL
	Urgert et al., 1997 [85] 114 mg diterpenes/day, 28 days	60 mg cafestol and 54 mg kahweol	−3 mg/dL
	Zock et al., 1990 [78] 1.3 g coffee lipids/day, 6 weeks	150 mg unsaponifiable lipids	−0.8 mg/dL (nearly unchanged)
No significant effect on HDL cholesterol	Van Rooij et al., 1995 [30] 1 g coffee oil (Arabica/day), 6 weeks	70 mg cafestol and 87 mg kahweol	No significant change
	Van Rooij et al., 1995 [30] 1 g coffee oil (Canephora/day), 6 weeks	39 mg cafestol and 1 mg kahweol	No significant change
	Boekschoten et al., 2005 [90] 2 mL coffee oil/day, 5 weeks	69 mg cafestol and 51 mg kahweol	No significant change
	Weusten-Van der Wouw et al., 1994 [38] 2 g coffee oil/day, 4 weeks	57 mg cafestol and 69 mg kahweol	No significant change
Rise in LDL cholesterol	Boekschoten et al., 2003 [86] 2 mL coffee oil/day, 5 weeks	69 mg cafestol and 51 mg kahweol	+27 mg/dL
	Weusten-Van der Wouw et al., 1994 [38] 2 g coffee oil/day, 4 weeks	57 mg cafestol and 69 mg kahweol	Increased (not further defined)
	Heckers et al., 1994 [88] 148 mg diterpenes/day	N/A	+42 mg/dL
	Urgert et al., 1997 [85] 64 mg cafestol/day, 28 days	64 mg cafestol	+22 mg/dL
	Urgert et al., 1997 [85] 114 diterpenes/day, 28 days	60 mg cafestol and 54 mg kahweol	+27 mg/dL
	Urgert et al., 1996 [77] 71 mg diterpenes/day, 24 weeks	38 mg cafestol and 33 mg kahweol	+19 mg/dL
	Van Rooij et al., 1995 [30] 1 g coffee oil (Arabica/day), 6 weeks	70 mg cafestol and 87 mg kahweol	+30 mg/dL
	Van Rooij et al., 1995 [30] 1 g coffee oil (Canephora/day), 6 weeks	39 mg cafestol and 1 mg kahweol	+17 mg/dL
	Zock et al., 1990 [78] 1.3 g coffee lipids/day, 6 weeks	150 mg unsaponifiable lipids	+33 mg/dL

If necessary, the unit was converted to mg/dL. N/A: data not available.

The term serum cholesterol contains three main components: HDL cholesterol, LDL cholesterol, and triglycerides [89]. HDL is often considered as “good” cholesterol, and values >40 mg/dL are desired in order to help prevent coronary heart diseases. The HDL values of healthy men are 30–65 mg/dL and 35–80 mg/dL for women [89]. The evaluated studies did not reveal any significant effects on HDL [30,38,56,76,78,85,86]. It should be added that the study review by Jee et al. showed no significant effect on HDL if six cups of unfiltered coffee are consumed [91]. Furthermore, Urgert et al. concluded that the effects of cafestol and kahweol are not equivalent: kahweol has less capacity than cafestol to interfere with lipid metabolism in humans [85].

**Figure 4:**
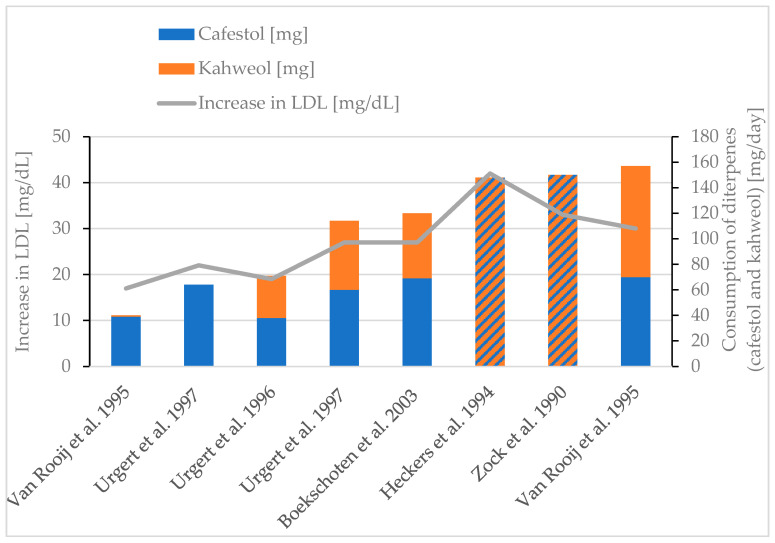
Increase in LDL. Review of six studies [30,77,78,85,86,88]. Striped bars (diagonal hatching): only diterpene content is given in the respective study [78,88].

Increased LDL cholesterol is connected to “hardening of the arteries” or atherosclerotic cardiovascular disease (ASCVD) [92]. Different studies have observed an increase in LDL cholesterol while consuming diterpenes [30,77,78,85,86,88] (Figure 4). The increase in LDL cholesterol roughly follows the diterpene consumption. For patients with a coronary heart disease, an LDL value of <70 mg/dL is desirable [89], meaning that an increase of up to 42 mg/dL could be considered a health hazard.

**Figure 5:**
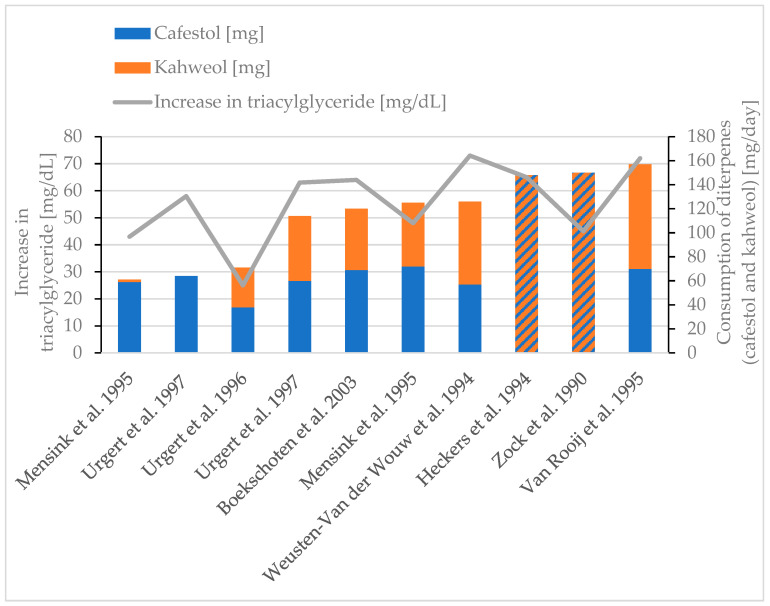
Increase in triacylglycerol. Review of eight studies [30,38,77,78,85,86,87,88]. Striped bars (diagonal hatching): only diterpene content is given in the respective study [78,88].

The triacylglyceride values rose [30,38,77,78,85,86,87,88] with higher diterpene consumption (Figure 5). The triacylglyceride parameter is recommended to be <200 mg/dL [89]. Two studies did not show any significant effects on triacylglycerides [30,76]. In contrast, Weusten-Van der Wouw et al. showed that only the consumption of a coffee beverage containing diterpenes leads to increasing serum cholesterol or triglyceride values. If a beverage does not contain diterpenes, no increase in these values is observed [38].

#### 3.7.3. Effects on Liver Enzymes

In contrast to the previous findings regarding serum lipids, it is more difficult to draw conclusions on liver enzymes due to the limited data available (Table 5). In the following, the current knowledge regarding the effects on alanine aminotransferase (ALT), aspartate aminotransferase (AST), alkaline phosphatase (ALP), y-glutamyl-transferase (GGT), and creatinine is investigated [30,38,77,85,90].

**Figure 6:**
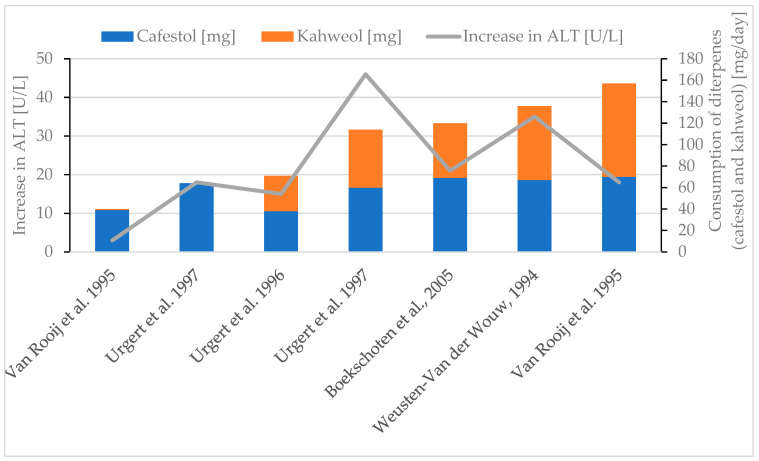
Increase in ALT. Review of five studies [30,38,77,85,90].

**Figure 7:**
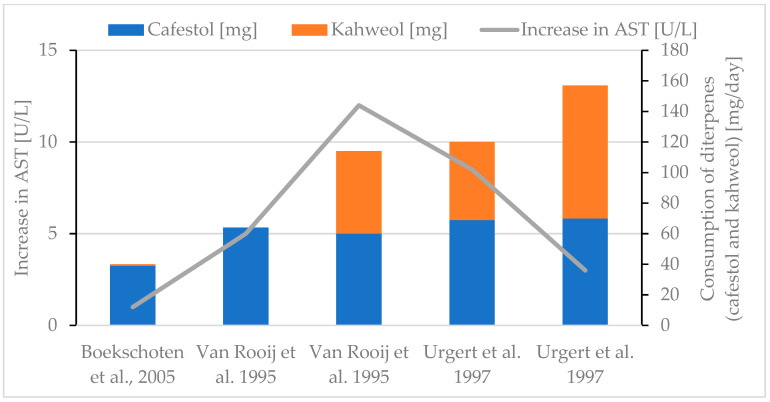
Increase in AST. Review of three studies [30,85,90].

**Figure 8:**
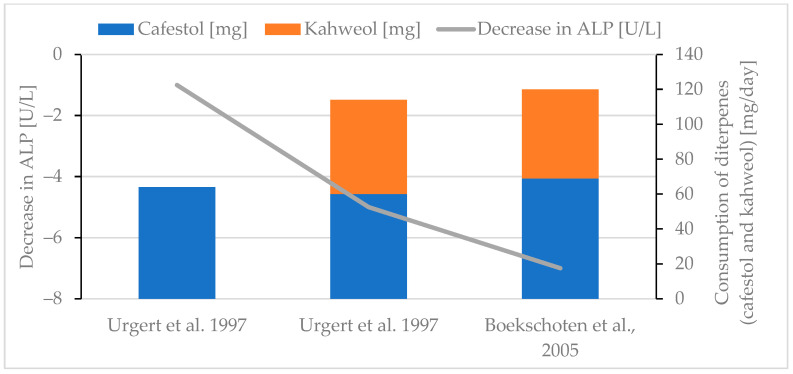
Decrease in ALP. Review of two studies [85,90].

Generally, an increase in the liver parameter ALT was observed if higher amounts of diterpenes were consumed (Figure 6) [30,38,77,85,90], although the effect was not as distinct as for the serum lipid parameters. The evaluation of the AST parameter leads to conflicting results, but the consumption of increasing amounts of diterpenes (especially after adding kahweol) seems to lead to decreasing AST levels (Figure 7) [30,85,90]. More data are needed for definitive conclusions. This contrasts with the results for ALT, whose increase follows more or less the diterpene intake (Figure 5) [30,38,77,85,90]. ALP appears to decrease with a higher diterpene intake (Figure 8) [77,85,90]. Several studies either report no significant effect [30] or a decrease [38,77,85,90] for the GGT parameter. A maximal decrease of 3 U/L for GGT was reported if 114 mg diterpenes/day was consumed [85]. With regard to the available literature, creatinine is reported to decrease as a result of diterpene consumption [38,85]. To date, there is no evidence to suggest that changes in liver enzyme levels are clinically relevant or would lead to drug-induced liver injury (DILI) [29].

#### 3.7.4. Effects on Other Parameters

In the evaluated studies, other different parameters were also investigated and will be roughly summarized in the following (Table 6). The consumption of cafestol and kahweol may lead to a decrease in amylase [90] as well as the lipoprotein(a) level [93]. An increase in the apolipoprotein B level (APO B) [30,56,76] is described as well as an increase in the serum lathosterol level (indicator of cholesterol synthesis) [76] and the 7α-hydroxy-4-cholesten-3-one level [90]. The evaluated literature describes no significant effect on apolipoprotein A-I (APO AI) [30,56,76], bile acid [90], bilirubin [90], the serum campesterol level (indicator of cholesterol absorption) [76], lactate dehydrogenase (LDH) [30,90], serum total and free triiodothyronine (T3), thyroxine (T4), and thyroid-stimulating hormone (TSH) [87], as well as body weight [30,77,86]. These parameters are unsuitable for the overall risk assessment of coffee oil.

### 3.8. Proposed Mechanism of Action of Cafestol

Cafestol, especially present in unfiltered coffee brews, is known as the most potent cholesterol-raising agent that may be present in the human diet. More research needs to be performed on this topic, as the mechanisms behind the cholesterol-increasing effect are only partly understood. The metabolic fate of cafestol and kahweol remains even less well-understood [29,62,71,90,94,95,96,97,98,99].

A mechanism was proposed that could explain the repression of bile acid synthesis, as well as the increase in detoxification and cholesterol by consuming cafestol present in unfiltered coffee. The mechanism was proposed using ApoE*3-Leiden mice, studying the nuclear hormone receptors farnesoid X receptor (FXR) and pregnane X receptor (PXR). There are two pathways proposed that lead to a reduced synthesis of bile acids: On the one hand, cafestol could activate FXR and PXR in the intestine, which induces FGF15. Via PXR, cafestol also induces Cyp3A11 and GSTµ1, resulting in increased detoxification. On the other hand, cafestol could activate FXR in the liver [95].

### 3.9. Potential Health Benefits of Cafestol and Kahweol

Beneficial effects are reported in the context of cafestol and kahweol consumption, especially via in vitro, in silico, and animal studies. Various studies are focusing on the possible anti-carcinogenic effects of diterpenes [83,100,101,102,103,104,105], e.g., against colorectal cancer [106], certain leukemia cell lines [107], head and neck cancer cells [108], colon cancer [88,109], or renal cancer [110]. Herein, the diterpenes might be potential anti-cancer agents, adjuvants to chemotherapeutic agents, or anti-angiogenetic agents [58,104,111,112].

This anti-carcinogenic effect could be due to inhibition of DNA damage, most likely due to the induction of phase II enzymes involved in carcinogen detoxification. This effect could also be caused by a reduction in the expression or inhibition of phase I enzyme activity responsible for carcinogen activation and the stimulation of intracellular antioxidant defense mechanisms [27,39,113,114]. This should be evaluated in the context of possible Maillard reaction products in coffee oil [10].

In contrast, Grubben et al. found no effect on potential biomarkers for colon cancer [115]. Due to their ability to form adducts, cafestol and kahweol show anti-genotoxic and chemoprotective effects against aflatoxin B1 (AFB1) and 7,12-dimethylbenz[*a*]anthracene (DMBA) [27,116]. Additionally, anti-inflammatory [99,103] and hepatoprotective effects [114] were examined. Furthermore, the two compounds are reported to play a positive role in the functioning of the immune system by increasing glutathione S-transferase enzyme activity during phase II biotransformation by the liver. Increased enzyme activity helps to remove toxic substances from the body to prevent unwanted reactions with other macromolecules [69,71,117,118,119,120]. It should be noted that green coffee oil does not show cytotoxic effects using concentrations up to 20 mg/mL [39].

### 3.10. Acute or Subacute Toxicity

Regarding, e.g., hematological and biochemical parameters, no acute toxicity of green coffee oil enriched with cafestol and kahweol was observed in rats by Oliveira et al., as the findings suggest that the LD50 is higher than the used coffee oil dosages of 2000 mg/kg (dose for acute study). Using dosages from 25 to 75 mg/kg for a subacute study (28 days), the highest dose revealed different effects, such as a decrease in body weight, serum glucose, and triacylglycerides and an increase in liver weight. The results of the subacute study could be explained by increased metabolism/detoxification once the organism was exposed to a new xenobiotic [121].

### 3.11. Further Remarks

It is advised that hypercholesterolemic patients avoid unfiltered coffee beverages and therefore the consumption of coffee oil [38,91]. This can be compared to the advice for hypercholesterolemic patients to follow a diet with a lower butter consumption [122]. Urgert et al. assumed that it is unlikely that taking oral contraceptives impacts the level of the lipoprotein(a) decrease in blood regarding coffee oil intake [93]. Whether other parameters could be affected by the use of contraceptives requires further research. No literature was found dealing, e.g., with the effects of coffee oil on unborn children, children, or adolescents. Furthermore, Urgert et al. observed that women tend to respond less to coffee diterpenes regarding effects on serum lipid levels than men do [85]. Variations in the response of blood parameters could be due to polymorphisms, e.g., genes encoding proteins involved in bile acid metabolism [86]. This topic is left for further research.

## 4. Discussion

Several animal and human studies were conducted in order to examine the effect of coffee oil or, more precisely, the diterpenes cafestol and kahweol. It is known that animal models such as hamsters, rats, gerbils, and non-human primates (Cebus, Rhesus, and African green monkeys) react differently than human models: humans, for example, react with a rise in serum cholesterol in response to cafestol and kahweol, in contrast to animal models, even with different dosages, modes of administration, or durations of the exposure [97,123].

However, it should be added that there are conflicting data in the literature; for example, some authors describe rats as a useful animal model to study the hypercholesterolemic effects of cafestol [124]. Only human studies from 1990 [78] to 2005 [90] have been used for this toxicological risk assessment, as these most closely resemble the response to coffee oil consumption. This risk assessment focuses on adverse health effects, while potential positive effects are not taken into consideration. Our systematic review revealed no human studies published after 2005.

In order to determine an acceptable coffee oil intake regarding the blood parameters total cholesterol, LDL, and triglycerides, a comparison with general coffee consumption or a general diet might be useful. As HDL remains nearly unchanged according to most of the studies, it is not taken into consideration for the assessment.

### 4.1. Comparison of Blood Parameter Changes Due to Coffee Oil with Other Foods

**Figure 9:**
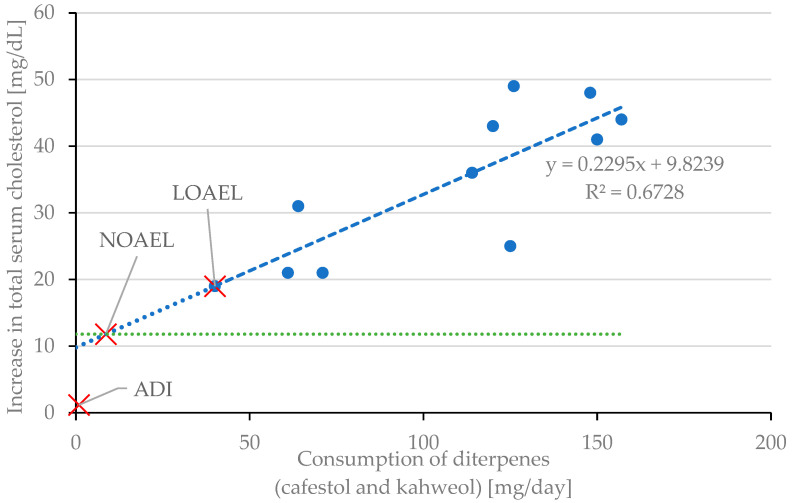
Determination of LOAEL, NOAEL, and ADI regarding the evaluated studies if linearity is assumed [30,38,77,78,85,86,87,88]. Blue dotted line: linear regression of the data points. Green dotted line: cholesterol level after coffee consumption [91].

Engel et al. published a trial regarding the changes in blood parameters if moderate amounts of butter are consumed versus the consumption of olive oil [122]. The consumption of 16.6 g of butter leads to an increase in the cholesterol level of 10.8 mg/dL. As described in the following, the increasing effect of butter or olive oil on blood parameters is comparable to the effect when unfiltered coffee is consumed: Jee et al. reviewed 14 published trials regarding coffee consumption in the period from 1985 to 1992. It was concluded that, on average, if six cups of unfiltered coffee are consumed, total cholesterol, LDL, and triacylglycerides increase by 11.8 mg/dL, 6.5 mg/dL, and 5.9 mg/dL, respectively, while HDL is hardly affected [91]. These findings are supported by this literature review regarding coffee oil (Table 4). Therefore, these parameters regarding coffee consumption are used in the following (11.8 mg/dL if six cups of coffee are consumed). Figure 9 summarizes the findings of all the evaluated studies. Based on these data, values for LOAEL, NOAEL, and ADI were estimated.

### 4.2. Determining LOAEL, NOAEL, and ADI of Cafestol and Kahweol

The biological variation in total cholesterol levels for humans averages 6.1% and can be as high as 11% [125]. Considering the general serum cholesterol level of a healthy human (which is around 220–280 mg/dL [89]), normal total cholesterol variation could be around 24 to 31 mg/dL. Therefore, the Lowest Observed Adverse Effect Level (LOAEL) of diterpene intake levels could be defined at 40 mg/day, based on the study of van Rooij et al. [30], observing the lowest effect on humans (Table 7). The No Observed Adverse Effect Level (NOAEL) may be assumed at about 9 mg/day of diterpene intake based on findings due to coffee consumption [91]. Considering an uncertainty factor of 10 for intra-human variability, an Acceptable Daily Intake (ADI) of 0.9 mg/day of diterpene intake may be deduced.

For precautionary public health protection, the authors suggest restricting additional coffee oil intake from other sources aside from coffee considering the proposed ADI.

## 5. Conclusions

Several studies regarding coffee oil consumption were recently conducted. In summary, the assessment based on the published data reveals that (i) the consumption of coffee oil contained in any type of prepared coffee appears to be safe because the homeostasis of lipid levels in the blood is not significantly affected, and (ii) a low consumption of coffee oil as such might be safe but would require a refined risk assessment considering the exposure levels of the intended food product, which must be provided for novel food approval procedures.

For a further risk assessment of coffee oil as a novel food, according to EFSA standards [19], only a few data are missing, e.g., the effects of coffee oil regarding allergies and the effects on risk groups, such as hypercholesterolemic patients, pregnant women, and children, have not yet been researched extensively.

## Figures and Tables

**Table 1:** General composition of coffee oil (lipids of green coffee) (reprinted without changes from Speer and Kölling-Speer, CC BY-NC 4.0 license) [9]).

Compounds	% Dry Matter [9]
Triacylglycerols	75.2
Esters of diterpene alcohols and fatty acids	18.5
Diterpene alcohols	0.4
Esters of sterols and fatty acids	3.2
Sterols	2.2
Tocopherols	0.04–0.06
Phosphatides	0.1–0.5
Tryptamine derivatives	0.6–1.0

**Table 2:** Typical cafestol and kahweol contents of different coffee brews (taken from [62]).

	Cafestol (mg/100 mL)	Kahweol (mg/100 mL)
Scandinavian boiled	0.5–8	0.7–10
Turkish/Greek	0.3–6.7	0.1–7.1
French press	1.5–3.7	1.7–5.3
Espresso	0.1–1.9	0.1–2.6
Filter	0–0.1	0–0.1

**Table 3:** Estimated maximum amount of the daily intake of the diterpenes cafestol/kahweol via coffee consumption in a worst-case assumption.

	Adult
Maximal daily intake of coffee [mL coffee/day]	1914
Maximal diterpene content of coffee [mg diterpenes/100 mL coffee]	18
Diterpene intake based on the max. coffee intake [mg diterpenes per day]	345 ^1^
Diterpene intake based on the max. coffee intake ^2^ [mg diterpenes/kg bw/day]	6

^1^ 345 mg of diterpenes is equal to about 153 mg of cafestol and 191 mg of kahweol. ^2^ For a human of 60 kg body weight.

**Table 5:** Liver enzymes: examples of effects in humans due to diterpene (cafestol/kahweol) or unfiltered coffee consumption.

Effect	Studies with Humans	Cafestol/Kahweol Content (per Day)	∆
Rise in ALT	Boekschoten et al., 2005 [90] 2 mL coffee oil/day, 5 weeks	69 mg cafestol and 51 mg kahweol	+21 U/L
	Weusten-Van der Wouw, 1994 [38] 2 g coffee oil/day, 4 weeks	57 mg cafestol and 69 mg kahweol	+35 U/L
	Van Rooij et al., 1995 [30] 1 g coffee oil (Arabica/day), 6 weeks	70 mg cafestol and 87 mg kahweol	+18 U/L
	Van Rooij et al., 1995 [30] 1 g coffee oil (Canephora/day), 6 weeks	39 mg cafestol and 1 mg kahweol	+3 U/L
	Urgert et al., 1997 [85] 64 mg cafestol/day, 28 days	64 mg cafestol	+18 U/L
	Urgert et al., 1997 [85] 114 diterpenes/day, 28 days	60 mg cafestol and 54 mg kahweol	+46 U/L
	Urgert et al., 1996 [77] 71 mg diterpenes/day, 24 weeks	38 mg cafestol and 33 mg kahweol	+15 U/L
Rise in AST	Boekschoten et al., 2005 [90] 2 mL coffee oil/day, 5 weeks	69 mg cafestol and 51 mg kahweol	+8.5 U/L
	Van Rooij et al., 1995 [30] 1 g coffee oil (Arabica/day), 6 weeks	70 mg cafestol and 87 mg kahweol	+3 U/L
	Van Rooij et al., 1995 [30] 1 g coffee oil (Canephora/day), 6 weeks	39 mg cafestol and 1 mg kahweol	+1 U/L
	Urgert et al., 1997 [85] 64 mg cafestol/day, 28 days	64 mg cafestol	+5 U/L
	Urgert et al., 1997 [85] 114 mg diterpenes/day, 28 days	60 mg cafestol and 54 mg kahweol	+12 U/L
	Urgert et al., 1996 [77] 71 mg diterpenes/day, 24 weeks	38 mg cafestol and 33 mg kahweol	Marginally increased (not further defined)
Decrease in GGT	Weusten-Van der Wouw, 1994 [38] 2 g coffee oil/day, 4 weeks	57 mg cafestol and 69 mg kahweol	Decreased (not further defined)
	Urgert et al., 1997 [85] 64 mg cafestol/day, 28 days	64 mg cafestol	−1 U/L
	Urgert et al., 1997 [85] 114 mg diterpenes/day, 28 days	60 mg cafestol and 54 mg kahweol	−3 U/L
	Urgert et al., 1996 [77] 71 mg diterpenes/day, 24 weeks	38 mg cafestol and 33 mg kahweol	Decreased (not further defined)
	Boekschoten et al., 2005 [90] 2 ml coffee oil/day, 5 weeks	69 mg cafestol and 51 mg kahweol	−1 U/L
Decrease in ALP	Urgert et al., 1997 [85] 64 mg cafestol/day, 28 days	64 mg cafestol	−1 U/L
	Urgert et al., 1997 [85] 114 mg diterpenes/day, 28 days	60 mg cafestol and 54 mg kahweol	−5 U/L
	Urgert et al., 1996 [77] 71 mg diterpenes/day, 24 weeks	38 mg cafestol and 33 mg kahweol. Maximum values from the 24-week study used	Decreased (not further defined)
	Boekschoten et al., 2005 [90] 2 mL coffee oil/day, 5 weeks	69 mg cafestol and 51 mg kahweol	−7 U/L
No effect on GGT	Van Rooij et al., 1995 [30] 1 g coffee oil (Arabica/day), 6 weeks	70 mg cafestol and 87 mg kahweol	No significant change
	Van Rooij et al., 1995 [30] 1 g coffee oil (Canephora/day), 6 weeks	39 mg cafestol and 1 mg kahweol	No significant change
Decrease in creatinine	Weusten-Van der Wouw, 1994 [38] 2 g coffee oil/day, 4 weeks	57 mg cafestol and 69 mg kahweol	Decreased (not further defined)
	Urgert et al., 1997 [85] 64 mg cafestol/day, 28 days	64 mg cafestol	−3 µmol/L
	Urgert et al., 1997 [85] 114 mg diterpenes/day, 28 days	60 mg cafestol and 54 mg kahweol	−8 µmol/L

If required, the unit of parameters given in the studies was converted to mg/dL.

**Table 6:** Examples of effects in humans due to diterpene (cafestol/kahweol) or unfiltered coffee consumption.

Effect	Studies with Humans	Cafestol/Kahweol Content (per day)	∆
Decrease in Lipoprotein(a) level	Urgert, 1997 [93] coffee oil daily, 4 weeks	85 mg cafestol and 103 mg kahweol	−4.8 mg/dL
Rise in 7a-Hydroxy-4-cholesten-3-one level	Boekschoten et al., 2005 [90] 2 mL coffee oil/day, 5 weeks	69 mg cafestol and 51 mg kahweol	+2.7 µg/L
No effect on APO A-I	Van Dusseldorp 1991 [76] (0.9 L unfiltered boiled coffee/day, 14 weeks)	N/A	No significant change
	Ahola et al., 1991 [56] (1 L unfiltered boiled coffee/day, 4 weeks)	N/A	No significant change
	Van Rooij et al., 1995 [30] 1 g coffee oil (Arabica/day), 6 weeks	70 mg cafestol and 87 mg kahweol	No significant change
	Van Rooij et al., 1995 [30] 1 g coffee oil (Canephora/day), 6 weeks	39 mg cafestol and 1 mg kahweol	No significant change
No effect on bile acid	Boekschoten et al., 2005 [90] 2 mL coffee oil/day, 5 weeks	69 mg cafestol and 51 mg kahweol	No significant change
No effect on bilirubin	Boekschoten et al., 2005 [90] 2 mL coffee oil/day, 5 weeks	69 mg cafestol and 51 mg kahweol	No significant change
Decrease in amylase	Boekschoten et al., 2005 [90] 2 mL coffee oil/day, 5 weeks	69 mg cafestol and 51 mg kahweol	−0.5 U/L
Increase in APO B	Van Dusseldorp 1991 [76] 0.9 L unfiltered boiled coffee/day, 14 weeks	N/A	+8.6 mmol/L +4412 g/L
	Ahola et al., 1991 [56] 1 L unfiltered boiled coffee/day, 4 weeks	N/A	+0.0001 mmol/L +0.05 g/L
	Van Rooij et al., 1995 [30] 1 g coffee oil (Arabica/day), 6 weeks	70 mg cafestol and 87 mg kahweol	+0.35 g/L
	Van Rooij et al., 1995 [30] 1 g coffee oil (Canephora/day), 6 weeks	39 mg cafestol and 1 mg kahweol	+0.17 g/L
No effect on serum campesterol level	Van Dusseldorp 1991 [76] 0.9 L unfiltered boiled coffee/day, 14 weeks	N/A	No significant change
Increase in serum lathosterol level	Van Dusseldorp 1991 [76] 0.9 L unfiltered boiled coffee/day, 14 weeks	N/A	N/A
No effect on LDH	Van Rooij et al., 1995 [30] 1 g coffee oil (Arabica/day), 6 weeks	70 mg cafestol and 87 mg kahweol	No significant change
	Van Rooij et al., 1995 [30] 1 g coffee oil (Canephora/day), 6 weeks	39 mg cafestol and 1 mg kahweol	No significant change
	Boekschoten et al., 2005 [90] 2 mL coffee oil/day, 5 weeks	69 mg cafestol and 51 mg kahweol	No significant change
No effect on T3, T4, and TSH	Mensink et al., 1995 [87] 2 g coffee oil /day, 3 weeks	72 mg cafestol and 53 mg kahweol	No significant change
	Mensink et al., 1995 [87] 2 g coffee oil /day, 3 weeks	59 mg cafestol (cafestol and O-methyl-cafestol) and 2 mg kahweol	No significant change
No effect on body weight	Van Rooij et al., 1995 [30] 1 g coffee oil (Arabica/day), 6 weeks	70 mg cafestol and 87 mg kahweol	No significant change
	Van Rooij et al., 1995 [30] 1 g coffee oil (Canephora/day), 6 weeks	39 mg cafestol and 1 mg kahweol	No significant change
	Urgert et al., 1996 [77] 71 mg diterpenes/day, 24 weeks	38 mg cafestol and 33 mg kahweol. Maximum values from the 24-week study used	No significant change (less than 0.5 kg/m^2^)
	Boekschoten et al., 2003 [86] 2 mL coffee oil/day, 5 weeks	69 mg cafestol and 51 mg kahweol	No significant change

**Table 7:** Summary of the toxicological thresholds of the diterpenes cafestol and kahweol.

Toxicological Threshold	Diterpene Intake (mg/day)	Coffee Oil Intake (mg/day) ^1^
LOAEL	40	10,000
NOAEL	9	2250
ADI	0.9	225

^1^ If it is assumed that coffee oil contains about 0.4% diterpenes (see Table 1).

## Data Availability

No new data were created or analyzed in this study.
